# A descriptive analysis of child-relevant systematic reviews in the Cochrane Database of Systematic Reviews

**DOI:** 10.1186/1471-2431-10-34

**Published:** 2010-05-20

**Authors:** Simon Bow, Jeffrey Klassen, Annabritt Chisholm, Lisa Tjosvold, Denise Thomson, Terry P Klassen, David Moher, Lisa Hartling

**Affiliations:** 1 Alberta Research Centre for Health Evidence, Department of Pediatrics, University of Alberta, Edmonton, Alberta, Canada; 2 Cochrane Child Health Field, Department of Pediatrics, University of Alberta, Edmonton, Alberta, Canada; 3 Stollery Children's Hospital, Edmonton, Alberta, Canada; 4 Clinical Epidemiology Program, Ottawa Hospital Research Institute, Ottawa, Canada

## Abstract

**Background:**

Systematic reviews (SRs) are considered an important tool for decision-making. There has been no recent comprehensive identification or description of child-relevant SRs. A description of existing child-relevant SRs would help to identify the extent of available child-relevant evidence available in SRs and gaps in the evidence base where SRs are required. The objective of this study was to describe child-relevant SRs from the Cochrane Database of Systematic Reviews (CDSR, Issue 2, 2009).

**Methods:**

SRs were assessed for relevance using pre-defined criteria. Data were extracted and entered into an electronic form. Univariate analyses were performed to describe the SRs overall and by topic area.

**Results:**

The search yielded 1666 SRs; 793 met the inclusion criteria. 38% of SRs were last assessed as up-to-date prior to 2007. Corresponding authors were most often from the UK (41%). Most SRs (59%) examined pharmacological interventions. 53% had at least one external source of funding. SRs included a median of 7 studies (IQR 3, 15) and 679 participants (IQR 179, 2833). Of all studies, 48% included only children, and 27% only adults. 94% of studies were published in peer-reviewed journals. Primary outcomes were specified in 72% of SRs. Allocation concealment and the Jadad scale were used in 97% and 25% of SRs, respectively. Adults and children were analyzed separately in 12% of SRs and as a subgroup analysis in 14%. Publication bias was assessed in only 14% of SRs. A meta-analysis was conducted in 68% of SRs with a median of 5 trials (IQR 3, 9) each. Variations in these characteristics were observed across topic areas.

**Conclusions:**

We described the methodological characteristics and rigour of child-relevant reviews in the CDSR. Many SRs are not up-to-date according to Cochrane criteria. Our study describes variation in conduct and reporting across SRs and reveals clinicians' ability to access child-specific data.

## Background

Systematic reviews (SRs) are considered the most comprehensive tool for decision-making by practitioners, policy-makers, and consumers. Systematic reviewers aim to identify all relevant data for a given question and synthesize the findings in a rigorous and transparent manner. The Cochrane Collaboration is identified as "the reliable source of evidence in healthcare" http://www.cochrane.org. One of the mechanisms through which Cochrane SRs are disseminated is the Cochrane Database of Systematic Reviews (CDSR) which contains 3916 completed SRs covering a broad range of therapeutic interventions (CDSR Issue 3, 2009).

There has been no recent comprehensive identification or description of the child-relevant SRs contained within the CDSR. A description of existing child-relevant SRs would help to identify the extent of available child-relevant evidence available in SRs and gaps in the evidence base where SRs are required. This may assist with prioritization of topics for synthesis within different topic areas. Further, a description of the methodological approaches used in child-relevant SRs would provide information on the rigour and consistency in the conduct of these reviews.

We set out to describe child-relevant SRs with respect to a number of key variables. These efforts provide the groundwork for other initiatives such as prioritization activities, methodological research, and ongoing tagging of child-relevant reviews within the CDSR.

## Methods

### Definition of child-relevant SR

For the purposes of this project we defined a child-relevant SR as one that intended to include children (0-18 years of age) or studied an intervention intended to improve the health and well-being of children (e.g., smoking control programs for family and caregivers [1], family-centered care for hospitalized children [2], parenting programs for psychosocial outcomes in adolescents [3]). Systematic reviews related to pregnancy were excluded except for studies on breastfeeding or nutritional supplements for the baby during pregnancy. These criteria are consistent with those of the Cochrane Child Health Field Trials Register.

### Search

A Research Librarian searched all years of the CDSR (Issue 2, 2009) using a pediatric search filter to identify child-relevant SRs. The search strategy is listed in Additional file 1.

### Screening

The records that were identified from the search underwent two phases of screening for inclusion. A screening algorithm was developed *a priori *(Additional file 2). One author screened the titles and abstracts of all reviews and classified them as "include", "exclude", or "unsure". When necessary, the full text of the review was retrieved to assess relevance. The included reviews were further classified by study population as follows: children (intended population was only children); children and adults (intended population was children and adults); unclear (intended population included children but upper age limit unclear); pregnancy (topic involved breastfeeding or nutritional supplements during pregnancy). All reviews labelled "exclude" were assessed by a second reviewer (LH) to ensure accuracy in study selection. Any discrepancies were reviewed by a clinician (TK). Reviews labelled "unclear" were assessed by a second reviewer (LH) to determine relevance to child health. Those that remained unclear were reviewed by a clinician (TK) who made the final decision regarding inclusion.

### Data extraction

We developed and pilot tested an electronic form for data extraction (form available from corresponding author on request). Data were extracted and entered directly onto the electronic form using Microsoft Excel. The variables extracted fell into three main categories: general characteristics (publication dates, country of corresponding authors, nature of interventions, external source of funding); characteristics of included studies (study designs sought and included, number of studies and participants, ages represented in primary studies, whether or not included studies were published in peer-reviewed journals); and methodological approaches (whether authors specified a primary outcome, approach to methodological quality assessment, approach to analysis, whether meta-analyses were conducted, proportion of trials [and reports] contributing to the largest meta-analysis, whether publication bias was assessed).

The country of corresponding authors was classified on indices of human development (high, medium or low as defined by the United Nations [http://hdr.undp.org/en/statistics/, accessed July 2009]) and income level (high, upper-middle, lower-middle, or low income according to the World Bank [http://web.worldbank.org/WBSITE/EXTERNAL/DATASTATISTICS/0,,contentMDK:20421402~pagePK:64133150~piPK:64133175~theSitePK:239419,00.html, accessed July 2009]). The nature of the interventions under comparison was classified as pharmacological or non-pharmacological based on a definition provided by Health Canada (http://www.hc-sc.gc.ca/dhp-mps/prodpharma/databasdon/terminolog-eng.php, accessed May 2009). Standard definitions were used to classify interventions as a natural health product (http://www.hc-sc.gc.ca/dhp-mps/prodnatur/index-eng.php, accessed June 2009) or a device (http://www.fda.gov/CDRH/DEVADVICE/312.html, accessed June 2009). Studies were classified as children only (all participants < 18 years of age), adult only (all participants > = 18 years of age), or mixed.

### Data analysis

Univariate analyses were conducted to describe the SRs and the primary studies they contained. The data were analyzed overall and within each of the relevant Cochrane Review Groups (CRG) from among The Cochrane Collaboration's 52 CRGs. Data were presented separately for CRGs containing more than 25 child-relevant reviews.

## Results

**Figure 1:**
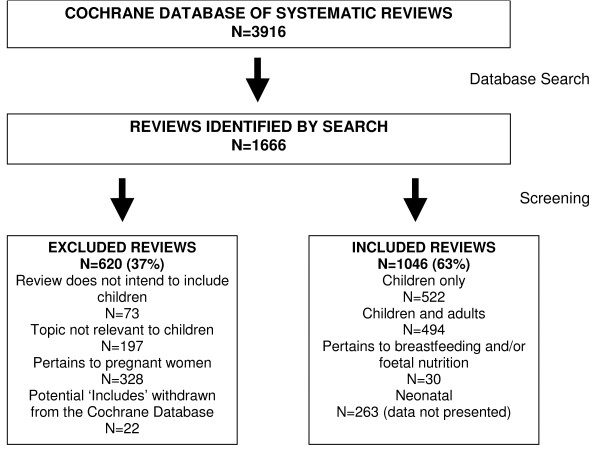
**Flow of systematic reviews through the screening process**.

**Figure 2:**
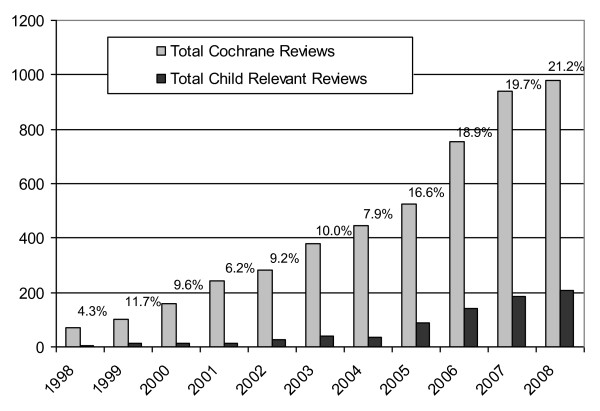
**Child-relevant reviews vs. all reviews in the Cochrane Database of Systematic Reviews (percentages represent child-relevant/all reviews)**.

Of the 3916 completed reviews in the CDSR, 1666 were identified through the search as potentially relevant to child health. Overall, 1046 met the inclusion criteria (Figure 1), and were included across 38 CRGs. The CRGs with the largest number of child-relevant reviews were: Neonatal (n = 253 representing 24% of all child-relevant reviews), Airways (n = 118; 11%), Acute Respiratory Infections (n = 70; 7%), Cystic Fibrosis and Genetic Disorders (n = 66; 6%), Infectious Diseases (n = 58; 5%), and Developmental, Psychosocial, and Learning problems (n = 49; 5%). Table 1 provides further details of the reviews by CRG, the proportions with children only and children and adult populations, and the proportion of child-relevant reviews to all reviews within each CRG. Figure 2 illustrates the increase of child-relevant reviews as a proportion of all reviews in the CDSR from 1998 to 2008. In the following sections we describe the child-relevant reviews; neonatal reviews were not included in the analysis as they have recently been described elsewhere [4].

**Table 1:** Child-relevant reviews by Cochrane Collaborative Review Group (CRG)

Cochrane Collaborative Review Group	Total completed reviews in CDSR	Total child-relevant reviews (percent of child relevant to all completed reviews)	Reviews intending to include only children (n)	Reviews intending to include both children and adults (n)
Neonatal	253	253 (100)	252	1
Airways	221	118 (53.4)	36	82
Acute Respiratory Infections	102	70 (68.6)	38	32
Cystic Fibrosis and Genetic Diseases	80	66 (82.5)	10	56
Infectious Diseases	94	58 (61.7)	9	49
Developmental, Psychosocial and Learning Problems	73	49 (67.1)	36	13
Oral Health	92	32 (33.7)	18	14
Epilepsy	50	30 (60.0)	6	24
Ear, Nose and Throat Disorders	55	28 (50.9)	12	16
Injuries	93	26 (28.0)	7	19
Pregnancy and Childbirth	359	26 (7.2)	1	25
Renal	74	22 (29.7)	8	14
HIV/AIDS	57	22 (38.6)	6	16
Neuromuscular Disease	76	21 (27.6)	2	19
Pain, Palliative and Supportive Care	113	20 (17.7)	4	16
Inflammatory Bowel Disease and Functional Bowel Disorders	50	19 (38.0)	3	16
Skin	42	16 (38.1)	4	12
Metabolic and Endocrine disorders	67	16 (23.9)	9	7
Anaesthesia	53	16 (30.2)	7	9
Heart	75	14 (18.7)	3	11
Musculoskeletal	117	14 (12.0)	5	9
Incontinence	60	12 (20.0)	8	4
Depression, Anxiety and Neurosis	104	12 (11.5)	5	7
Wounds	63	12 (19.0)	1	11
Eyes and Vision	75	10 (13.3)	8	2
Tobacco Addiction	48	9 (18.8)	4	5
Consumers and Communication	27	9 (33.3)	3	6
Gynaecological Cancer	68	8 (11.8)	0	8
Hepato-Biliary	107	6 (5.6)	1	5
Drugs and Alcohol	50	5 (10)	3	2
Colorectal Cancer	58	5 (8.6)	0	5
Movement Disorders	44	5 (11.4)	2	3
Upper Gastrointestinal and Pancreatic Diseases	41	5 (12.2)	3	2
Childhood Cancer	3	3 (100)	2	1
Peripheral Vascular Diseases	75	3 (4.0)	3	0
Effective Practice and Organization of Care	58	2 (3.5)	1	1
Schizophrenia	135	2 (1.5)	1	1
Haematological	20	2 (10.0)	1	1
TOTAL	3916	1046 (26.7)	522	524

### General characteristics of child-relevant reviews (Additional file 3)

The median dates of protocol and initial publication were 2002 and 2004, respectively. The median number of years between publication of protocol and review was 2 (IQR 1, 3). This varied across review groups, ranging from 1 year for Cystic Fibrosis and Genetics (IQR 1, 2), Developmental, Psychosocial, and Learning Problems (IQR 1, 2) and Injuries (IQR 0, 2) to 3 years (IQR 2, 4) for Oral Health. The median date for when reviews were 'last assessed as up-to-date' was 2007; 38% of SRs were last assessed as up-to-date prior to 2007.

The corresponding authors were most often from the UK (41.1%) followed by the rest of Europe (14.8%), North America (14.0%), Australia (14.0%), Asia (8.7%), Africa (4.9%), and South America (2.5%). The vast majority of reviews were produced in countries with indices of high income (85.6%) and high human development (88.4%). The countries classified as middle and low human development were most often represented in reviews produced by the Infectious Diseases Group.

The majority of reviews examined pharmacological interventions (59.0%) according to the Health Canada definition, which included drugs (52.2%), vaccines (3.6%), or natural health products (8.0%). The largest portion of non-pharmacological reviews (16.9% of total) consisted of educational, behavioural, psychological, policy, or legislative interventions. A small proportion of reviews (6.1%) compared pharmacological and non-pharmacological interventions. The nature of the intervention varied across CRGs reflecting the different topic areas, e.g., the Oral Health, the Developmental, Psychosocial and Learning Problems, and the Injuries Groups more often evaluated non-pharmacological interventions.

Approximately half of the reviews (52.5%) had at least one external source of funding, though many had multiple sources. The Infectious Diseases Group received external funding for over 90% of their child-relevant reviews. The most common sources of external funding were government (48.6%), foundation (15.4%), and Cochrane (12.6%).

### Characteristics of studies included in child-relevant reviews (Additional file 4)

The majority of reviews intended to include only randomized controlled trials (RCTs) (54.2%), while some searched for RCTs and other designs (most often quasi-RCTs) (45.4%) and very few searched only for non-RCTs (0.4%). In actuality, 71.6% of reviews included RCTs only, 27.1% included RCTs and other designs, and 1.3% included only non-RCTs. This varied substantially across CRGs, reflecting the nature of evidence available across different topics and types of interventions.

The median number of trials included in a review was 7 (inter-quartile range [IQR]: 3, 15). This varied across groups ranging from 3 (IQR: 1, 6.75) for Cystic Fibrosis to 10 (IQR: 6, 17.75) for Infectious Diseases. Seventy-four reviews (9.3%) had no relevant trials. This also varied between groups, with 2 (2.9%) in Acute Respiratory Infections to 14 (21.2%) in Cystic Fibrosis.

Children-only studies made up approximately half of the included studies (47.5%). Adult-only studies made up 26.9% of included studies and children-adult mixed studies made up 14.6%. The remainder of studies did not indicate a mean age or range of ages for participants. The median number of participants included in each review was 679 (IQR 179, 2833).

Overall, 9248 of studies (94.6%) included in the reviews were published in peer-reviewed journals.

### Methodological approaches in child-relevant reviews (Additional file 5)

Reviewers specified a primary outcome in 72.4% of reviews. This number varied substantially between groups, from 26.9% in Injuries to 90.0% in Infectious Diseases.

Allocation concealment was the most common approach to assessing methodological quality, used in 96.9% of reviews. Among 5171 included studies, allocation concealment was adequate in 28.8%, inadequate in 7.5% and unclear in 62.2%. The Jadad scale was used in 181 (25.1%) reviews; this also varied substantially from 1.7% in Infectious Diseases to 63.6% in Airways.

Children were analyzed separately in 52 (11.5%) reviews that included both children and adults. Additionally, subgroup analyses for adults and children were performed in 63 (13.9%) reviews. Subgroup analyses were also performed within children in 35 (5.3%) reviews.

Publication bias was formally assessed in 97 (12.2%) reviews; it was assessed graphically in 90 (92.8%) and statistically in 32 (33.0%) reviews. In 158 (22.0%) reviews, publication bias was mentioned but was not assessed. The majority of reviews (63.8%) did not mention publication bias.

Meta-analyses were conducted in 484 reviews (68.3%). In those reviews that conducted a meta-analysis, a median of 5 trials were included in the largest meta-analysis conducted (IQR 3, 9). The median percentage of included studies that contributed to the largest meta-analysis in each review was 50% (IQR 33, 78).

## Discussion

The principal outcome of this study was the characterization of child-relevant SRs that are currently available in the Cochrane Database of Systematic Reviews. This descriptive analysis provides information on the extent of evidence synthesis on child-relevant topics as well as variation in methodological characteristics and rigour across the reviews. The register compiled through this effort provides a basis to identify gaps in the evidence base where reviews or updates are needed and to assist with prioritization for the production or updating of reviews within different topic areas. The register will also help identify priority areas for primary research in children. Further, ongoing identification and tagging of child-relevant SRs will facilitate access to pediatric-specific data.

Over the ten years studied, there has been a steady increase in the number and proportion of child-relevant reviews in the CDSR. This finding is inconsistent with the observation that the volume of adult trials is growing at a faster rate than pediatric trials [5]. One of the key variables that we extracted was the number of trials within each child-relevant review that involved children only, adults only, or mixed adult and children populations. We found that less than half (47.4%) of studies included in child-relevant reviews were conducted solely in children. Moreover, only 25.2% of reviews that included both children and adults conducted separate analyses (11.4%) or subgroup analyses (13.8%) to distinguish between the results of the two groups. This figure may in part reflect the fact that separate or subgroup analyses were not deemed necessary in some situations (e.g., studies involving older adolescents and adults where there is no developmental or physiological basis for differences in response to a particular intervention). The extent of separate or subgroup analyses for children varied across Review Groups which also may reflect different situations where there may or may not be reason to expect a difference in effect. Nevertheless, previous work has suggested that there may be insufficient evidence specific to children for certain topic areas [6]. This is particularly problematic in situations where the safety and efficacy of interventions for children may differ from adults due to variations in developmental physiology, disease pathophysiology, or developmental pharmacokinetics and pharmacodynamics [7]. Systematic reviewers should be considering separate or subgroup analyses for children and discuss the applicability of the evidence for age groups that may show differential effects.

Several additional observations can be made regarding the extent of evidence available for child-relevant reviews. Overall, 9.3% of reviews found no relevant studies. This represents an important portion of child-relevant reviews and suggests that there is a need for primary research in a variety of areas. The proportion of reviews with no relevant studies varied across Review Groups and was particularly high (>20%) for the Cystic Fibrosis and Genetic Disorders and the Developmental, Psychosocial, and Learning Disorders Groups. Further, the median number of studies per review was 7 (IQR 3, 15) which is consistent with an analysis of Cochrane reviews published in 2004 [8]. As many authors stated, this often provides insufficient data to attain significant results in meta-analyses, conduct subgroup analyses, or assess for publication bias. Finally, on average only half of relevant studies contributed to the largest meta-analysis in each review. Over-reliance on the results of meta-analyses is problematic as most meta-analyses will reflect only a portion of the available evidence. Research has demonstrated that the magnitude of effect decreases as the proportion of relevant trials contributing to the meta-analysis increases [9].

One of the key goals of The Cochrane Collaboration is to ensure that available evidence is up-to-date http://www.cochrane.org. To this end, The Cochrane Collaboration has implemented a policy that authors update their reviews every two years [10]. We found a median date of 2007 for when reviews were 'Last Assessed as Up-to-Date,' suggesting that the majority of reviews have been updated according to Cochrane policy. However, 38% of the relevant reviews were last assessed as up-to-date prior to 2007 and are therefore considered out-of-date by Cochrane standards. This proportion varied across Review Groups. Additionally, 22 of the reviews that we had originally identified as relevant to the register were subsequently found to be withdrawn from The Cochrane Library and could not be included in our analysis. While there is ongoing work on the appropriate timing of updates [11-14], mechanisms are needed to ensure that child-relevant reviews are in fact providing the most up-to-date accurate information for decision-makers. This presents a challenge for reviewers in terms of the time and resources required for updates, as well as the end-users of the reviews or those wanting to synthesize the reviews (e.g., in overviews of reviews) as a basis for decision-making.

A further goal of The Cochrane Collaboration is to be global in its scope and meet the health information needs of people worldwide. Consistent with a previous descriptive analysis of SRs published in 2004 [8], we found that an overwhelming majority of studies had corresponding authors in countries with a high rating on indices of human development and income level. This variable is simply a proxy for the applicability of topics to countries with different indices of human development and income level. For instance, many of the reviews with a corresponding author from these countries may in fact be relevant to lower income/development countries and may also have co-authors from these countries. There was interesting variation across Review Groups. For example, authors from the Airways and the Cystic Fibrosis and Genetic Disorders groups were primarily from high income regions (UK, Australia, North America) whereas those in the Acute Respiratory Infections and especially the Infectious Diseases Groups were more evenly distributed worldwide, and more often included authors from Africa and Asia. This likely reflects the conditions of most interest, and perhaps the highest prevalence, in developing countries.

Approximately 40% of reviews examined non-pharmacological interventions, which is consistent with a sample of Cochrane reviews published in 2004 [8]. The most frequently examined non-pharmacological interventions were educational, behavioural, psychological, policy or legislative interventions. This reflects the usefulness of reviews within the CDSR to a variety of end-users including a range of practitioners and policy-makers. The variety of interventions examined in reviews may also explain some of the variability observed in the types of study designs included.

One of the strengths of The Cochrane Collaboration is that it includes methodological experts worldwide and produces cutting-edge methods for the conduct of systematic reviews. The Cochrane Handbook represents state of the art systematic review methods [10]. Further, Cochrane reviews have been found to have better reporting quality than paper-based reviews [8,15]. Despite this, we found substantial variation in the conduct of reviews across the Review Groups. For example, over 27% of reviews did not specify a primary outcome and this proportion varied substantially across Review Groups, ranging from 10 to 73%. Specification of primary outcomes should be done at the protocol stage. Peer-reviewed, published protocols are required for Cochrane reviews; the PRISMA group that develops guidelines for the reporting of systematic reviews is now advocating registration of all SR protocols to enhance "transparency and accountability" [16].

The approach to assessment of methodological quality also varied across reviews. Allocation concealment was most commonly assessed; this is consistent with the recommended Cochrane methods prior to 2008 after which time the Risk of Bias (RoB) tool was introduced. Despite long-standing caution against the use of scales for quality assessment [17], the Jadad scale was often used, particularly within certain Review Groups. Finally, a very small proportion of reviews assessed for publication bias which is consistent with a previous sample [8]. Most reviews did not mention publication bias at any point; however, some authors stated that an assessment was intended, but was not done, likely due to an insufficient number of included studies. Our findings suggest a potential for publication bias in the reviews, as we found that a high percentage of the included studies (94.6%) were published in peer-reviewed journals. Previous research suggests that published studies tend to present positive findings. For example, in a study of abstracts presented at a major pediatric research meeting, only 60% were subsequently published and those published were more likely to report positive findings [18].

Approximately half of the reviews received no funding from external sources. This varied substantially across review groups, with over 90% of reviews in the Infectious Diseases Group receiving external funding compared to only 20% in the Epilepsy Group. The time and resources required to conduct a methodologically rigorous SR are substantial. Lack of funding will seriously hamper efforts to synthesize evidence in child health. While we did not collect data regarding funding for updates, it is likely that funding is less frequently available for updating reviews. This will be an important obstacle to achieving the Cochrane's mission of ensuring that evidence for decision-making is up-to-date.

This work provides a solid foundation for future research in two key areas. First, the data we have collected will serve as a baseline to examine changes and standardization in review methods over time, as well as the availability of child specific evidence. Second, this work provides a basis for methodological research investigating bias at the level of SRs, as well as trials (e.g., through meta-epidemiological methods). Empirical evidence of bias specific to child-relevant research will result in a better understanding of biases and how they operate in this context.

### Limitations of this study

The main limitation we encountered resulted from inconsistent or lack of reporting by review authors for key variables, confirming previous findings that there is room for improvement in reporting of SRs [15]. For example, the number of participants included in the reviews was inconsistently reported. Sometimes the overall number was reported in the abstract or beginning of the results section. Other times, the data extractors had to rely on the information in the Characteristics of Included Studies table. Review authors did not always specify whether the number cited corresponded to participants recruited, randomized, or analyzed. Due to these and other caveats, the total number of participants cited in our data is likely smaller than those that were actually included in all reviews. The other variable that was particularly problematic was whether the included studies included children-only, adult-only, or mixed populations. Often this detail was not reported for each included study. We used a strict cut-off of 18 years to differentiate study populations; therefore, some studies with older adolescents would be categorized as mixed populations but the actual participants may be relatively homogeneous in terms of physiology or development. We chose inclusion criteria that were consistent with the Cochrane Child Health Field Trials Register which excludes all pregnancy studies except those evaluating breastfeeding or nutritional supplements. There are numerous other interventions administered during pregnancy that may affect infant outcomes; however, these were not captured in this descriptive analysis and may be an interesting focus for future work. Moreover, we may have missed reviews of interventions that do not directly involve children but are intended to improve their health.

## Conclusions

We have described the evidence available from child-relevant reviews in the CDSR. Children-only studies represented less than half of the studies included in child-relevant reviews. There is a need for more evidence specific to children, particularly when efficacy or safety may differ across age groups. There is wide variation in methods across the Cochrane Review Groups. Standardization in methods based on empirical evidence should be encouraged. Many reviews are considered out-of-date according to Cochrane standards. The majority of reviews were conducted in high income countries, and therefore may not reflect the health priorities of lower- or middle-income countries. This information will serve to inform the conduct and focus of future SRs and primary research in child health. Moreover, the register compiled through this effort will serve as a basis for methodological research to understand biases in reviews and primary studies.

## Competing interests

The authors declare that they have no financial competing interests. LT, DT, TPK, DM and LH are members of The Cochrane Collaboration.

## Authors' contributions

SB screened systematic reviews for inclusion, performed data extraction and data analysis, and drafted portions of the manuscript. JK performed data extraction and data analysis. AC performed data extraction and data analysis. LT conducted the search and assisted with defining inclusion criteria. DT provided background material and contributed to study methodology. DM provided methodological advice. TK provided methodological and clinical advice. LH developed the protocol, oversaw all aspects of the project, drafted portions of the manuscript, and coordinated submission and revisions of the completed manuscript. All authors provided critical feedback on the manuscript and approved the final manuscript.

## Pre-publication history

The pre-publication history for this paper can be accessed here:

http://www.biomedcentral.com/1471-2431/10/34/prepub

## Supplementary Material

Additional file 1 **Search strategy**. Search strategy for identification of child-relevant systematic reviews in the Cochrane Database of Systematic Reviews Click here for file

Additional file 2 **Screening algorithm**. Screening algorithm for inclusion of reviews in Child Health Systematic Review Register Click here for file

Additional file 3 **General characteristics of child-relevant reviews**. Table describing general characteristics of child-relevant reviews, overall and by review groups with more than 25 child-relevant reviews Click here for file

Additional file 4 **Characteristics of studies included in child-relevant reviews**. Table describing characteristics of studies included in child-relevant reviews, overall and by review groups with more than 25 child-relevant reviews Click here for file

Additional file 5 **Methodological approaches in child-relevant reviews**. Table describing methodological approaches in child-relevant reviews, overall and by review groups with more than 25 child-relevant reviews Click here for file
