# Supplementary material for: A descriptive analysis of child-relevant systematic reviews in the Cochrane Database of Systematic Reviews
Source: BMC Pediatr. 2010 May 20;10:34. doi: 10.1186/1471-2431-10-34 (PMC2881081; doi:10.1186/1471-2431-10-34)
Supplement: Additional file 1 — Search strategy. Search strategy for identification of child-relevant systematic reviews in the Cochrane Database of Systematic Reviews [file 1471-2431-10-34-S1.DOC]

Additional file 1. Search strategy

Cochrane Database of Systematic Reviews (Issue 2 2009)

Search conducted: 28Apr09

ID Search

#1 (Infant* OR infancy OR Newborn* OR Baby* OR Babies OR Neonat* OR Preterm* OR Prematur* OR Postmatur* OR Child* OR Schoolchild* OR School age* OR Preschool* OR Kid or kids OR Toddler* OR Adoles* OR Teen* OR Boy* OR Girl* OR Minors* OR Pubert* OR Pubescen* OR Prepubescen* OR Pediatric* OR Paediatric* OR Peadiatric* OR Nursery school* OR Kindergar* OR Primary school* OR Secondary school* OR Elementary school* OR High school* OR Highschool*):ti,ab,kw in Cochrane Reviews

#2 "Types of Participants" NEAR/8 ("any age" or "children" OR "infants" OR "neonates" or "teenagers" or "adolescents" or "all ages" or "18 years or younger") in Cochrane Reviews

#3 (#2 OR #1)
