# Supplementary figures and images for: A descriptive analysis of child-relevant systematic reviews in the Cochrane Database of Systematic Reviews
Source: BMC Pediatr. 2010 May 20;10:34. doi: 10.1186/1471-2431-10-34 (PMC2881081; doi:10.1186/1471-2431-10-34)

Screening algorithm for inclusion of reviews in Child Health Systematic Review Register


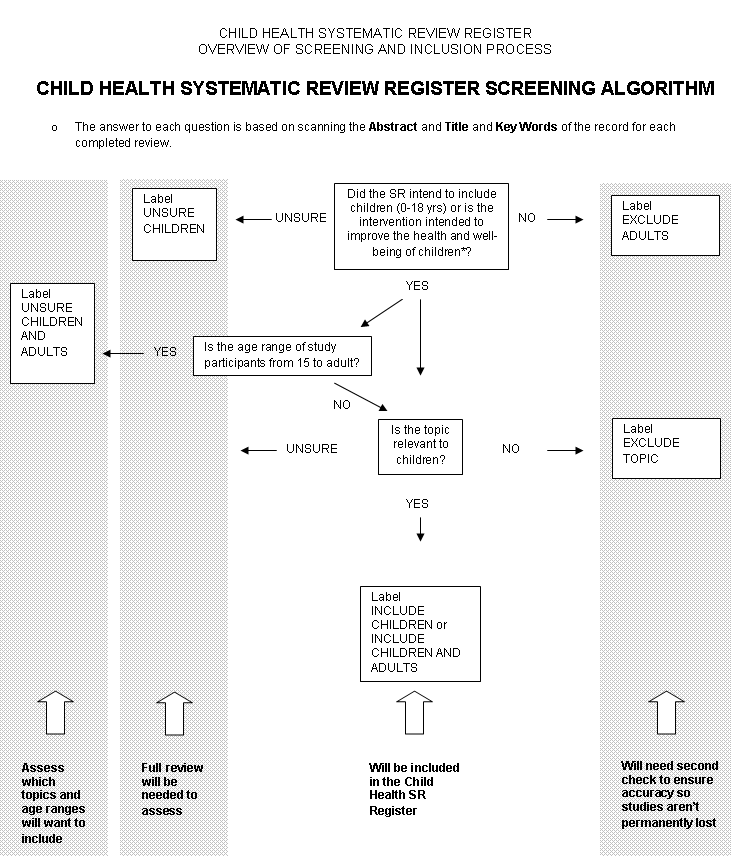


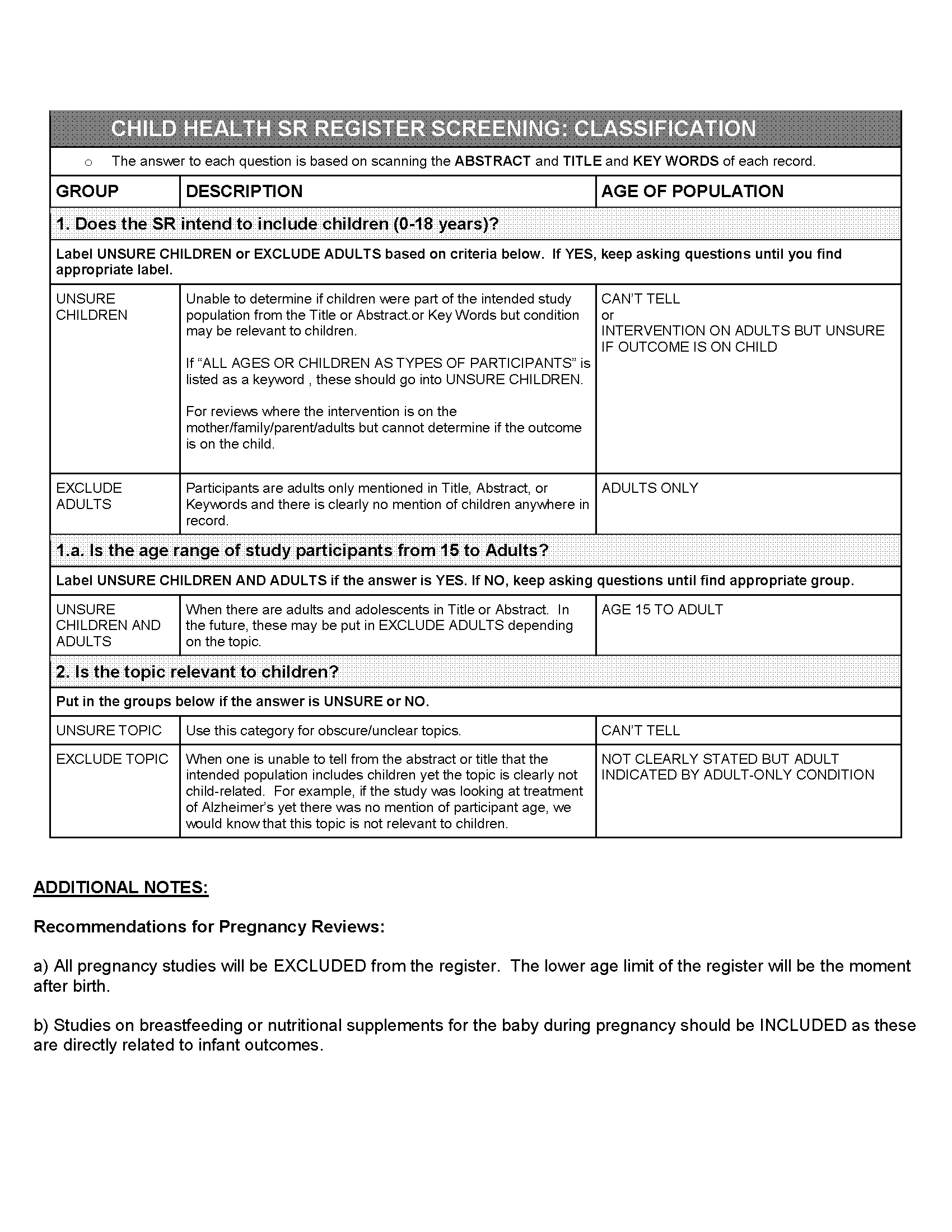

Supplement: Additional file 2 — Screening algorithm. Screening algorithm for inclusion of reviews in Child Health Systematic Review Register [file 1471-2431-10-34-S2.DOC]
