# Supplementary material for: A descriptive analysis of child-relevant systematic reviews in the Cochrane Database of Systematic Reviews
Source: BMC Pediatr. 2010 May 20;10:34. doi: 10.1186/1471-2431-10-34 (PMC2881081; doi:10.1186/1471-2431-10-34)
Supplement: Additional file 3 — General characteristics of child-relevant reviews. Table describing general characteristics of child-relevant reviews, overall and by review groups with more than 25 child-relevant reviews [file 1471-2431-10-34-S3.DOC]

| General characteristics of child-relevant reviews, overall and by review groups with more than 25 child-relevant reviews | | | | | | | | | | |
| --- | --- | --- | --- | --- | --- | --- | --- | --- | --- | --- |
|  | Overall  N= 793 | Airways  N=118 | Acute Respiratory Infections  N=70 | Cystic Fibrosis and Genetic Disorders  N=66 | Infectious Diseases  N=58 | Developmental, Psychosocial, and Learning Problems  N=49 | Oral Health  N=32 | Epilepsy  N=30 | Ear, Nose and Throat Disorders  N=28 | Injuries  N=26 |
| *Publication characteristics* | | | | | | | | | | |
| Year protocol published (median) | 2002 | 2000 | 2002/2003 | 2001 | 2001/2002 | 2003 | 2002 | 2000 | 2003 | 2003 |
| Year review published (median) | 2004 | 2003 | 2005 | 2003 | 2004 | 2006 | 2005 | 2002 | 2005 | 2004 |
| Number of years between publication of protocol and review (median [IQR]) | 2 (1,3) | 2 (1,2) | 2 (1,3) | 1 (1,2) | 1.5 (1,3) | 1 (1,2) | 3 (2,4) | 2 (1,2) | 1.5 (1,3) | 1 (0,2) |
| Year last assessed as up to date (median) | 2007 | 2007 | 2006 | 2008 | 2006 | 2005 | 2005 | 2007 | 2006 | 2007 |
| *Country Classification of corresponding author: income level (World Bank: http://www.worldbank.org/)*, n (% total) | | | | | | | | | | |
| High income | 679 (85.6) | 113 (95.8) | 48 (68.6) | 62 (93.9) | 30 (51.7) | 47 (95.9) | 29 (90.6) | 26 (86.7) | 25 (89.3) | 26 (100) |
| Upper middle income | 42 (5.3) | 2 (1.7) | 7 (10.0) | 2 (3.0) | 3 (5.2) | 1 (2.0) | 3 (9.4) | 1 (3.3) | 3 (10.7) | 0 (0) |
| Lower middle income | 66 (8.3) | 3 (2.5) | 15 (21.4) | 2 (3.0) | 21 (36.2) | 1 (2.0) | 0 (0.0) | 3 (10.0) | 0 (0.0) | 0 (0) |
| Low income | 6 (0.8) | 0 (0.0) | 0 (0.0) | 0 (0.0) | 4 (6.9) | 0 (0.0) | 0 (0.0) | 0 (0.0 | 0 (0.0) | 0 (0.0) |
| *Nature of intervention: classification 1* , n (% total) | | | | | | | | | | |
| Pharmacological | 468 (59.0) | 92 (78.0) | 64 (91.4) | 47 (71.2) | 49 (84.5) | 7 (14.3) | 13 (40.6) | 26 (86.7) | 13 (46.4) | 5 (19.2) |
| Non-pharmacological | 277 (34.9) | 23 (19.5) | 5 (7.1) | 14 (21.2) | 5 (8.6) | 41 (83.7) | 19 (59.4) | 4 (13.3) | 12 (42.9) | 16 (80.8) |
| Both pharmacological and non-pharmacological | 48 (6.1) | 3 (2.5) | 1 (1.4) | 5 (7.6) | 4 (6.9) | 1 (2.0) | 0 (0.0) | 0 (0.0) | 3 (10.7) | 0 |
| *Nature of intervention: classification 2* , n (% total) | | | | | | | | | | |
| Drug | 431 (52.2) | 81 (67.5) | 46 (64.8) | 38 (54.3) | 39 (61.9) | 6 (12.0) | 14 (40.0) | 26 (86.7) | 14 (50.0) | 5 (19.2) |
| Vaccine | 30 (3.6) | 4 (3.3) | 8 (11.3) | 2 (2.9) | 11 (17.5) | 0 (0.0) | 0 (0.0) | 0 (0.0) | 0 (0.0) | 0 (0.0) |
| Natural Health Product | 66 (8.0) | 5 (4.2) | 9 (12.7) | 10 (14.3) | 3 (4.8) | 2 (4.0) | 1 (2.9) | 1 (3.3) | 1 (3.6) | 0 (0.0) |
| Surgical/clinical | 76 (9.2) | 4 (3.3) | 4 (5.6) | 9 (12.9) | 4 (6.3) | 1 (2.0) | 9 (25.7) | 0 (0.0) | 5 (17.9) | 3 (11.5) |
| Educational/ behavioural/ psychological/ policy/ legislative | 140 (16.9) | 10 (8.3) | 0 (0.0) | 3 (4.3) | 0 (0.0) | 33 (66.0) | 2 (5.7) | 2 (6.7) | 0 (0.0) | 16 (61.5) |
| Device | 39 (4.7) | 5 (4.2) | 1 (1.4) | 3 (4.3) | 3 (4.8) | 0 (0.0) | 9 (25.7) | 1 (3.3) | 3 (10.7) | 2 (7.7) |
| Other | 44 (5.3) | 11 (9.2) | 3 (4.2) | 5 (7.1) | 3 (4.8) | 8 ( 16.0) | 0 (0.0) | 0 (0.0) | 5 (17.9) | 0 (0.0) |
| *External source of funding,* n (% total) | | | | | | | | | | |
| Not stated | 203 (25.6) | 15 (12.7) | 21 (30.0) | 39 (59.1) | 3 (5.2) | 8 (16.3) | 3 (9.4) | 22 (73.3) | 17 (60.7) | 3 (11.5) |
| No | 174 (21.9) | 24 (20.3) | 23 (32.9) | 9 (13.6) | 2 (3.4) | 7 (14.3) | 15 (46.9) | 2 (6.7) | 2 (7.1) | 6 (23.1) |
| Yes | 416 (52.5) | 79 (66.9) | 26 (37.1) | 18 (27.3) | 53 (91.4) | 34 (69.4) | 14 (43.8) | 6 (20.0) | 9 (32.1) | 17 (65.4) |
| Government | 281 (48.6) | 58 (48.7) | 14 (46.7) | 12 (50.0) | 52 (68.4) | 28 (54.9) | 10 (62.5) | 4 (33.3) | 4 (36.4) | 12 (63.2) |
| Cochrane | 73 (12.6) | 9 (7.6) | 6 (20.0) | 4 (16.7) | 4 (5.3) | 6 (11.8) | 4 (25.0) | 0 (0.0) | 4 (36.4) | 3 (15.8) |
| Foundation | 89 (15.4) | 28 (23.5) | 6 (20.0) | 1 (4.2) | 2 (2.6) | 10 (19.6) | 1 (6.3) | 0 (0.0) | 1 (9.1) | 2 (10.5) |
| Disease-specific | 16 (2.8) | 11 (9.2) | 0 (0.0) | 1 (4.2) | 0 (0.0) | 0 (0.0) | 0 (0.0) | 1 (8.3) | 0 (0.0) | 0 (0.0) |
| Hospital | 16 (2.8) | 5 (4.2) | 1 (3.3) | 0 (0.0) | 0 (0.0) | 0 (0.0) | 0 (0.0) | 1 (8.3) | 0 (0.0) | 1 (3.3) |
| Industry | 8 (1.4) | 0 (0.0) | 0 (0.0) | 0 (0.0) | 1 (1.3) | 0 (0.0) | 1 (6.3) | 1 (8.3) | 0 (0.0) | 0 (0.0) |
| Other | 95 (16.4) | 8 (6.7) | 3 (10.0) | 6 (25.0) | 17 (22.4) | 7 (13.7) | 0 (0.0) | 5 (41.7) | 2 (18.2) | 1 (5.3) |
