# Supplementary material for: A descriptive analysis of child-relevant systematic reviews in the Cochrane Database of Systematic Reviews
Source: BMC Pediatr. 2010 May 20;10:34. doi: 10.1186/1471-2431-10-34 (PMC2881081; doi:10.1186/1471-2431-10-34)
Supplement: Additional file 4 — Characteristics of studies included in child-relevant reviews. Table describing characteristics of studies included in child-relevant reviews, overall and by review groups with more than 25 child-relevant reviews [file 1471-2431-10-34-S4.DOC]

| Characteristics of studies included in child-relevant reviews, overall and by review groups with more than 25 child-relevant reviews | | | | | | | | | | |
| --- | --- | --- | --- | --- | --- | --- | --- | --- | --- | --- |
|  | Overall  N= 793 | Airways  N=118 | Acute Respiratory Infections  N=70 | Cystic Fibrosis and Genetic Disorders  N=66 | Infectious Diseases  N=58 | Developmental, Psychosocial and Learning Problems  N=49 | Oral Health  N=32 | Epilepsy  N=30 | Ear, Nose and Throat Disorders  N=28 | Injuries  N=26 |
| *Study Designs* | | | | | | | | | | |
| RCTs only (intended), n (% total) | 430 (54.2) | 101 (85.6) | 45 (64.3) | 23 (34.8) | 32 (55.2) | 19 (38.8) | 8 (25.0) | 17 (56.7) | 26 (92.9) | 11 (42.3) |
| RCTs only (actual), n (% of included studies) | 515 (71.6) | 96 (94.1) | 45 (66.2) | 44 (84.6) | 41 (73.2) | 24 (61.5) | 16 (55.2) | 18 (62.1) | 22 (95.7) | 14 (53.8) |
| RCTs and other designs (intended), n (% total) | 360 (45.4) | 17 (14.4) | 25 (35.7) | 43 (65.2) | 26 (44.8) | 30 (61.2) | 24 (75.0) | 13 (43.3) | 2 (7.1) | 14 (53.8) |
| RCTs and other designs (actual), n (% of included studies) | 195 (27.1) | 6 (5.9) | 23 (33.8) | 8 (15.4) | 15 (26.8) | 15 (38.5) | 13 (44.8) | 11 (37.9) | 1 (4.3) | 7 (26.9) |
| Non-RCTs (intended), n (% total) | 3 (0.4) | 1 (1.0) | 0 (0.0) | 0 (0.0) | 0 (0.0) | 0 (0.0) | 0 (0.0) | 0 (0.0) | 0 (0.0) | 1 (3.9) |
| Non-RCTs (actual), n (% of included studies) | 9 (1.3) | 1 (1.0) | 0 (0.0) | 0 (0.0) | 0 (0.0) | 1 (2.6) | 0 (0.0) | 0 (0.0) | 0 (0.0) | 4 (15.4) |
| *Studies and participants* | | | | | | | | | | |
| Number of studies included (median, IQR) | 7 (3,15) | 7 (2,20.25) | 7.5 (4,16.75) | 3 (1,6.75) | 10 (6,17.75) | 5 (2,9) | 4 (3,16) | 5 (2.5,10.75) | 9 (5,11) | 5 (3,18) |
| Reviews with no relevant studies, n, (% of reviews in group) | 74 (9.3) | 16 (13.6) | 2 (2.9) | 14 (21.2) | 2 (3.4) | 10 (20.4) | 3 (9.4) | 1 (3.3) | 5 (17.9) | 1 (3.8) |
| Child only studies [% of total number of studies] | 47.5 | 35.3 | 73.1 | 35.7 | 51.4 | 89.9 | 86.4 | 29.0 | 51.2 | 57.2 |
| Adult only studies [% of total number of studies] | 26.9 | 40.3 | 14.9 | 15.9 | 12.1 | 5.4 | 6.7 | 31.6 | 21.7 | 19.1 |
| Mixed child and adult studies [% of total number of studies] | 14.6 | 21.0 | 5.6 | 38.7 | 29.9 | 4.4 | 4.1 | 35.2 | 12.8 | 13.0 |
| Number of participants included (median, IQR) | 679  (179,2833) | 529  (159,3131.5) | 1731  (419.5,3464) | 145.5  (12.5,350.5) | 2377.5 (793.5,5554) | 362  (35,1106) | 353  (120,3233) | 907  (393.25, 1098.25) | 789  (369.5, 1007.5) | 3796  (178, 12303) |
| *Publication Characteristics* | | | | | | | | | | |
| Included studies published in peer-reviewed journals  n, (% of total included studies) | 9248 (94.6) | 1481 (90.9) | 876 (96.8) | 265 (80.8) | 746 (95.9) | 340 (92.6) | 499 (98.0) | 185 (95.9) | 202 (99.5) | 330 (87.8) |
