# Supplementary material for: A descriptive analysis of child-relevant systematic reviews in the Cochrane Database of Systematic Reviews
Source: BMC Pediatr. 2010 May 20;10:34. doi: 10.1186/1471-2431-10-34 (PMC2881081; doi:10.1186/1471-2431-10-34)
Supplement: Additional file 5 — Methodological approaches in child-relevant reviews. Table describing methodological approaches in child-relevant reviews, overall and by review groups with more than 25 child-relevant reviews [file 1471-2431-10-34-S5.DOC]

| Methodological approaches in child-relevant reviews, overall and by review groups with more than 25 child-relevant reviews | | | | | | | | | | |
| --- | --- | --- | --- | --- | --- | --- | --- | --- | --- | --- |
|  | Overall  N= 793 | Airways  N=118 | Acute Respiratory Infections  N=70 | Cystic Fibrosis and Genetic Disorders  N=66 | Infectious Diseases  N=58 | Developmental, Psychosocial, and Learning Problems  N=49 | Oral Health  N=32 | Epilepsy  N=30 | Ear, Nose and Throat Disorders  N=28 | Injuries  N=26 |
| *Outcomes* | | | | | | | | | | |
| Reviewers specified one or more primary outcomes, n (%) | 574 (72.4) | 99 (83.9) | 48 (68.6) | 26 (39.4) | 52 (90.0) | 20 (40.8) | 26 (81.3) | 19 (63.3) | 24 (85.7) | 7 (26.9) |
| *Assessment of methodological quality in SR’s with included studies* | | | | | | | | | | |
| Jadad, n (%) | 181 (25.1) | 75 (63.6) | 28 (40.0) | 7 (10.6) | 1 (1.7) | 1 (2.0) | 1 (3.4) | 1 (3.4) | 2 (8.7) | 0 (0.0) |
| Allocation concealment, n (%) | 699 (96.9) | 102 (86.4) | 68 (97.1) | 52 (78.8) | 56 (96.6) | 38 (77.6) | 29 (100) | 28 (96.6) | 22 (95.7) | 17 (68.0) |
| Components, n (%) | 522 (65.8) | 44 (37.3) | 47 (67.1) | 28 (42.4) | 56 (96.6) | 32 (65.3) | 29 (100) | 29 (100) | 21 (91.3) | 8 (30.8) |
| Other tool, n (%) | 53 (6.7) | 3 (2.5) | 9 (12.9) | 1 (1.5) | 0 (0.0) | 3 (6.1) | 0 (0.0) | 0 (0.0) | 1 (4.3) | 8 (30.8) |
| *Analysis* | | | | | | | | | | |
| Children analyzed separately in reviews with both child and adult participants, n (%) | | | | | | | | | | |
| Yes | 52 (11.5) | 14 (18.9) | 7 (25.9) | 0 (0.0) | 5 (11.9) | 0 (0.0) | 4 (17.4) | 0 (0.0) | 4 (26.7) | 3 (18.8) |
| No | 402 (88.5) | 60 (81.1) | 20 (74.1) | 45 (100.0) | 37 (88.1) | 13 (100.0) | 19 (82.6) | 22 (73.3) | 11 (73.3) | 13 (81.3) |
| N/A | 339 (42.7) | 44 (37.3) | 43 (61.4) | 21 (31.8) | 16 (27.6) | 36 (73.5) | 9 (28.1) | 8 (26.7) | 13 (46.4) | 10 (38.5) |
| Subgroup analyses for children in reviews with both child and adult participants, n (%) | | | | | | | | | | |
| Yes | 63 (13.9) | 27 (36.5) | 4 (14.8) | 1 (2.2) | 7 (16.7) | 0 (0.0) | 0 (0.0) | 2 (6.7) | 0 (0.0) | 1 (6.3) |
| No | 391 (86.1) | 47 (63.5) | 23 (85.2) | 44 (97.8) | 35 (83.3) | 13 (100.0) | 23 (100.0) | 20 (66.7) | 15 (100.0) | 15 (93.8) |
| N/A | 339 (42.7) | 44 (37.3) | 43 (61.4) | 21 (31.8) | 16 (27.6) | 36 (73.5) | 9 (28.1) | 8 (26.7) | 13 (46.4) | 10 (38.5) |
| Subgroup analyses within children in any review with children included, n (%) | | | | | | | | | | |
| Yes | 35 (5.3) | 4 (4.1) | 10 (15.4) | 1 (2.0) | 2 (3.6) | 0 (0.0) | 4 (14.3) | 0 (0.0) | 1 (4.5) | 2 (9.1) |
| No | 628 (94.7) | 94 (95.9) | 55 (84.6) | 49 (98.0) | 53 (96.4) | 39 (100.0) | 24 (85.7) | 28 (100.0) | 21 (95.5) | 20 (90.9) |
| N/A | 130 (16.4) | 20 (16.9) | 5 (7.1) | 16 (24.2) | 3 (5.2) | 10 (20.4) | 4 (12.5) | 2 (6.7) | 6 (21.4) | 4 (15.4) |
| Publication bias assessed, n (%) | | | | | | | | | | |
| Yes | 97 (12.2) | 17 (17.0) | 14 (20.6) | 2 (3.8) | 16 (28.6) | 2 (5.1) | 9 (31.0) | 0 (0.0) | 3 (13.0) | 5 (21.7) |
| No | 163 (20.6) | 37 (37.0) | 9 (13.2) | 9 (17.3) | 8 (14.3) | 21 (53.8) | 8 (27.6) | 0 (0.0) | 4 (17.4) | 7 (30.4) |
| Unclear | 459 (57.9) | 46 (46.0) | 45 (66.2) | 41 (78.8) | 32 (57.1) | 16 (41.0) | 12 (41.4) | 29 (100%) | 16 (69.6) | 11 (47.8) |
| N/A | 74 (9.3) | 18 (15.3) | 2 (2.9) | 14 (21.2) | 2 (3.4) | 10 (20.4) | 3 (9.4) | 1 (3.3) | 5 (17.9) | 3 (11.5) |
| Meta-analysis conducted in those reviews with included studies, n (%) | 483 (68.3) | 81 (68.6) | 50 (71.4) | 28 (42.4) | 49 (84.5) | 23 (46.9) | 14 (48.3) | 21 (70.0) | 8 (34.8) | 12 (46.2) |
| Number of studies contributing to meta-analysis (median, IQR) | 5 (3,9) | 6 (3,11) | 5 (3,10.75) | 2 (2,3) | 5 (3,9) | 5 (3.5,7) | 8 (3,15.5) | 4 (3,5) | 3 (2,7) | 6.5 (3,10.25) |
| Percentage of included studies contributing to meta-analysis (in reviews that conducted a meta-analysis) (median, IQR) | 50.0 (33.3,77.8) | 46.7 (33.3,66.7) | 66.7 (39.0,88.8) | 50.0 (27.7,67.9) | 58.6 (29.6,83.3) | 55.8 (38.7,66.7) | 54.4 (37.6,77.1) | 83.3 (44.4,100) | 43.8 (30.3,77.8) | 47.7 (35.2,80.0) |
